# Correction: Auxiliary subunits of the CKAMP family differentially modulate AMPA receptor properties

**DOI:** 10.7554/eLife.42543

**Published:** 2018-10-11

**Authors:** Paul Farrow, Konstantin Khodosevich, Yechiam Sapir, Anton Schulmann, Muhammad Aslam, Yael Stern-Bach, Hannah Monyer, Jakob von Engelhardt

Farrow P, Khodosevich K, Sapir Y, Schulmann A, Aslam M, Stern-Bach Y, Monyer H, von Engelhardt J. 2015. Auxiliary subunits of the CKAMP family differentially modulate AMPA receptor properties. *eLife* **4**:e09693. doi: 10.7554/eLife.09693.

Published 1, December 2015

In the Materials and Methods subsection "In situ hybridization" the sequences of radiolabeled oligodeoxyribonucleotide probes used for CKAMP52 and CKAMP59 were mixed up. These are the correct sequences:

Ckamp52ins1 = 5’AATGTCAGCCAGAGCCCTGTGGATGTTCATCTCTCGCGGA

Ckamp59ins1 = 5’GCGGCATAGCACGCCAGTCGAGGTTGGAGGGCTTCATGGTGTT

We apologize for the mistake.

The article has been corrected accordingly.

