# Supplementary material for: Auxiliary subunits of the CKAMP family differentially modulate AMPA receptor properties
Source: eLife. 2015 Dec 1;4:e09693. doi: 10.7554/eLife.09693 (PMC4733035; doi:10.7554/eLife.09693)
Supplement: Supplementary file 1. [file elife-09693-supp1.zip › Supplementary File 1.docx]

Supplementary file 1A - table 1

| Macropatches of oocytes (mean ± SEM) | | | | | | |
| --- | --- | --- | --- | --- | --- | --- |
|  | GluA1 | n | +CKAMP39 | n | +CKAMP52 | n |
| Amplitude  (pA) | 280 ± 63 | 12 | 153 ± 41 | 10 | 196 ± 44 | 15 |
| τ_deact_  (ms) | 2.5 ± 0.1 | 12 | 3.2 ± 0.2 | 9 | 2.6 ± 0.1 | 11 |
| τ_des_  (ms) | 3.3 ± 0.2 | 9 | 3.5 ± 0.1 | 10 | 3.2 ± 0.1 | 15 |
| ss/peak current  (%) | 0.6 ± 0.3 | 9 | 0.5 ± 0.1 | 10 | 1.5 ± 0.4 | 15 |
| τ_recovery_  (ms) | 235 ± 12 | 11 | 788 ± 95**** | 5 | 279 ± 10* | 12 |
|  | | | | | | |
|  | GluA2 | n | +CKAMP39 | n | +CKAMP52 | n |
| Amplitude  (pA) | 413 ± 115 | 14 | 209 ± 107 | 17 | 469 ± 111 | 15 |
| τ_deact_  (ms) | 2.2 ± 0.1 | 14 | 4.9 ± 0.3**** | 15 | 5.4 ± 0.5*** | 11 |
| τ_des_  (ms) | 7.9 ± 0.3 | 11 | 4.9 ± 0.4**** | 17 | 6.0 ± 0.2*** | 15 |
| ss/peak current  (%) | 3.8 ± 0.5 | 11 | 1.2 ± 0.2** | 17 | 3.3 ± 0.4 | 15 |
| τ_recovery_  (ms) | 72 ± 7 | 8 | 126 ± 9*** | 5 | 55 ± 3* | 10 |

Supplementary file 1B - table 2

| Glutamate EC50 (mean ± SEM) | | | | | | | |
| --- | --- | --- | --- | --- | --- | --- | --- |
| GluA1 | n | GluA1 + CKAMP39 | n | GluA1 + CKAMP44 | n | GluA1 + CKAMP52 | n |
| 14.9 ± 0.6 | 5 | 8.0 ± 0.4** | 5 | 3.6 ± 0.2*** | 5 | 4.8 ± 0.2**** | 4 |
| GluA2 | n | GluA2 + CKAMP39 | n | GluA2 + CKAMP44 | n | GluA2 + CKAMP52 | n |
| 27.5 ± 0.6 | 6 | 10.7 ± 0.7** | 4 | 11.2 ± 0.3** | 4 | 1.7 ± 0.1*** | 4 |
| CTZ EC50 (mean ± SEM) | | | | | | | |
| GluA1 | n | GluA1 + CKAMP39 | n | GluA1 + CKAMP44 | n | GluA1 + CKAMP52 | n |
| 16 ± 1 | 5 | 46 ± 2* | 4 | 22 ± 1 | 4 | 16 ± 1 | 4 |
| GluA2 | n | GluA2 + CKAMP39 | n | GluA2 + CKAMP44 | n | GluA2 + CKAMP52 | n |
| 6 ± 1 | 4 | 19 ± 3* | 5 | 15 ± 1* | 5 | 6 ± 1 | 3 |

Supplementary file 1C - table 3

| HEK293 cells (median [IQR]) | | | | | | | | |
| --- | --- | --- | --- | --- | --- | --- | --- | --- |
|  | GluA1 | n | +CKAMP39 | n | +CKAMP52 | n | +CKAMP59 | n |
| Amplitude  (pA) | 666  [445-1539] | 39 | 295***  [111-591] | 48 | 880  [403-1811] | 29 | 827  [487-1309] | 37 |
| τ_deact_  (ms) | 1.7  [1.5-2.2] | 39 | 1.9  [1.6-2.3] | 47 | 1.9  [1.7-2.6] | 37 | 1.9  [1.6-2.0] | 29 |
| τ_des_  (ms) | 3.4  [3.0-5.0] | 43 | 3.0  [2.4-4.1] | 36 | 3.8  [2.9-4.9] | 35 | 3.7  [3.0-6.3] | 27 |
| ss/peak current  (%) | 0.7  [0.5-1.7] | 43 | 0.8  [0.4-1.4] | 35 | 1.6*  [0.7-3.2] | 35 | 0.7  [0.1-1.7] | 27 |
| τ_recovery_  (ms) | 99  [78-114] | 27 | 952***  [228-1371] | 29 | 96  [76-127] | 25 | 95  [84-113] | 16 |
|  | | | | | | | | |
|  | GluA2 | n | +CKAMP39 | n | +CKAMP52 | n | +CKAMP59 | n |
| Amplitude  (pA) | 643  [143-1429] | 64 | 269*  [106-715] | 52 | 204**  [65-698] | 59 | 296*  [75-843] | 56 |
| τ_deact_  (ms) | 1.7  [1.5-2.2] | 64 | 1.6  [1.5-1.9] | 59 | 2.1**  [1.8-2.9] | 59 | 1.7  [1.3-2.0] | 56 |
| τ_des_  (ms) | 7.4  [6.6-9.3] | 59 | 4.4***  [3.6-5.8] | 42 | 7.8  [6.0-9.4] | 49 | 7.7  [6.3-9.4] | 47 |
| ss/peak current  (%) | 1.9  [1.2-4.1] | 59 | 0.5***  [0.2-1.1] | 42 | 5.0*  [2.2-8.2] | 49 | 2.0  [0.7-3.4] | 47 |
| τ Recovery  (ms) | 22  [15-40] | 22 | 78**  [39-142] | 25 | 16  [8-21] | 20 | 25  [10-49] | 22 |

Supplementary file 1D - table 4

| Quantification of total and surface GluA1 in HEK293 cells (mean ± SD) | | | | | | | | |
| --- | --- | --- | --- | --- | --- | --- | --- | --- |
|  | GluA1 | n | +CKAMP39 | n | +CKAMP52 | n | +CKAMP59 | n |
| total GluA1  (% of control) | 100 ± 13 | 9 | 37 ± 5**** | 9 | 67 ± 12**** | 9 | 54 ± 13**** | 9 |
| surface GluA1  (% of control) | 100 ± 16 | 9 | 24 ± 6**** | 9 | 99± 16 | 9 | 57 ± 11**** | 9 |
| ratio surface/total  (% of control) | 100 ± 12 | 9 | 64 ± 19 | 9 | 154 ± 52** | 9 | 109 ± 27 | 9 |
| Quantification of total and surface GluA2 in HEK293 cells (mean ± SD) | | | | | | | | |
|  | GluA2 | n | +CKAMP39 | n | +CKAMP52 | n | +CKAMP59 | n |
| total GluA2  (% of control) | 100 ± 6 | 9 | 52 ± 10**** | 9 | 77 ± 6**** | 9 | 38 ± 3**** | 9 |
| surface GluA2  (% of control) | 100 ± 6 | 9 | 32 ± 6**** | 9 | 64 ± 8**** | 9 | 21 ± 2**** | 9 |
| ratio surface/total  (% of control) | 100 ± 9 | 9 | 62 ± 8**** | 9 | 83 ± 11** | 9 | 55 ± 7**** | 9 |
